# Thousands of CpGs Show DNA Methylation Differences in ACPA-Positive Individuals

**DOI:** 10.3390/genes12091349

**Published:** 2021-08-29

**Authors:** Yixiao Zeng, Kaiqiong Zhao, Kathleen Oros Klein, Xiaojian Shao, Marvin J. Fritzler, Marie Hudson, Inés Colmegna, Tomi Pastinen, Sasha Bernatsky, Celia M. T. Greenwood

**Affiliations:** 1 PhD Program in Quantitative Life Sciences, Interfaculty Studies, McGill University, Montréal, QC H3A 1E3, Canada; yixiao.zeng@mail.mcgill.ca; 2 Lady Davis Institute for Medical Research, Jewish General Hospital, Montréal, QC H3T 1E2, Canada; kaiqiong.zhao@mail.mcgill.ca (K.Z.); kathleen.klein@mail.mcgill.ca (K.O.K.); marie.hudson@mcgill.ca (M.H.); 3 Department of Epidemiology, Biostatistics and Occupational Health, McGill University, Montréal, QC H3A 1A2, Canada; 4 Digital Technologies Research Centre, National Research Council Canada, Ottawa, ON K1A 0R6, Canada; xiaojian.shao@nrc-cnrc.gc.ca; 5 Cumming School of Medicine, University of Calgary, Calgary, AB T2N 1N4, Canada; fritzler@ucalgary.ca; 6 Department of Medicine, McGill University, Montréal, QC H4A 3J1, Canada; ines.colmegna@mcgill.ca (I.C.); sasha.bernatsky@mcgill.ca (S.B.); 7 Division of Rheumatology, Jewish General Hospital, Montréal, QC H3T 1E2, Canada; 8 Division of Rheumatology, McGill University, Montréal, QC H3G 1A4, Canada; 9 Department of Human Genetics, McGill University, Montréal, QC H3A 0C7, Canada; tomi.pastinen@mcgill.ca; 10 Center for Pediatric Genomic Medicine, Children’s Mercy, Kansas City, MO 64108, USA; 11 Gerald Bronfman Department of Oncology, McGill University, Montréal, QC H4A 3T2, Canada

**Keywords:** rheumatoid arthritis, anti-citrullinated protein antibody positivity, targeted bisulfite sequencing, DNA methylation, differentially methylated cytosines, differentially methylated regions, cell adhesion

## Abstract

High levels of anti-citrullinated protein antibodies (ACPA) are often observed prior to a diagnosis of rheumatoid arthritis (RA). We undertook a replication study to confirm CpG sites showing evidence of differential methylation in subjects positive vs. negative for ACPA, in a new subset of 112 individuals sampled from the population cohort and biobank CARTaGENE in Quebec, Canada. Targeted custom capture bisulfite sequencing was conducted at approximately 5.3 million CpGs located in regulatory or hypomethylated regions from whole blood; library and protocol improvements had been instituted between the original and this replication study, enabling better coverage and additional identification of differentially methylated regions (DMRs). Using binomial regression models, we identified 19,472 ACPA-associated differentially methylated cytosines (DMCs), of which 430 overlapped with the 1909 DMCs reported by the original study; 814 DMRs of relevance were clustered by grouping adjacent DMCs into regions. Furthermore, we performed an additional integrative analysis by looking at the DMRs that overlap with RA related loci published in the GWAS Catalog, and protein-coding genes associated with these DMRs were enriched in the biological process of cell adhesion and involved in immune-related pathways.

## 1. Introduction

Rheumatoid arthritis (RA) is considered a chronic, systemic, inflammatory autoimmune disease characterized by synovial inflammation. RA diagnosis relies on recognition of clinical characteristics (including symmetrical polyarthritis), and is often supported by the presence of anti-citrullated protein antibodies (ACPA) in serum [1]. ACPAs are highly specific for RA, and ACPA-positivity usually predicts a more severe disease course [2]. RA risk is determined by known genetic and environmental factors; however, their role in explaining heterogeneity of symptoms and progression is still unclear [3,4]. DNA methylation is in fact a key mechanism that integrates genetic and environmental causes of disease. Aberrant DNA methylation has been shown to mediate genotype and smoking interaction in the development of ACPA-positive RA [5], and with specific patterns in different cell types of RA patients [6]. It has been implicated in the abnormal proliferation and invasiveness of fibroblast-like synoviocytes [7,8], although it is still not known if specific methylation signatures could be useful in predicting RA disease course.

We have previously shown differentially methylated cytosines (DMCs) according to ACPA levels [9] in an age- and sex-matched subset of CARTaGENE individuals (www.cartagene.qc.ca (blood samples obtained by July 2013; sequencing data accessed on 30 April 2016)), a cohort and biobank of over 40,000 subjects sampled from regions of Quebec, Canada. Methylation was measured with a targeted custom capture bisulfite sequencing method [10] that assessed approximately 4.8 million CpGs in regions known to be relevant for immune regulation [11]. This article presents the results of a replication study in a new subset of individuals sampled from the same cohort (blood samples obtained by May 2018; sequencing data accessed on 11 December 2018). Although methylation data were obtained using the same sequencing platform, updated library constructions and protocols targeted immune-related regions of the genome with more accuracy and achieved higher read depths. With the rich data from the replication study, associations between methylation and ACPA status are available at a larger set of CpGs with improved precision, so that we cannot only validate earlier results, but also identify new methylation loci.

Furthermore, to evaluate the implications of identified epigenetic modifications for RA risk, we integrate our results with previously-published genome-wide association studies (GWAS) for RA. We demonstrate overlap, at the gene level, between the signals identified through our bisulfite sequencing experiments and the loci demonstrating strong genetic associations with RA. In addition, we explore the biological function of enriched gene ontology (GO) terms, as well as implicated immune-related pathways.

## 2. Results

Throughout the paper, we will refer to two datasets: “Dataset 1” is the one generated for the initial, previously reported study [9], and “Dataset 2” is the replication study that we are now reporting for the first time.

### 2.1. Subjects in the Replication Dataset

The selection of subjects for custom methylC-capture sequencing (MCC-seq) for Dataset 2 was similar to Dataset 1 (see Methods Section 5.1). In this replication study, 120 selected samples from CARTaGENE participants with extreme ACPA levels (highly positive or negative) in serum were sequenced. Cell-type composition information was missing in 8/120 samples, so 112 samples were retained for association analysis. Furthermore, 8/112 self-reported a diagnosis of RA and were analyzed separately. Of the remaining 104 samples, 50 were labeled as ACPA-positive (with optical density of ACPA over 60) and 54 were ACPA-negative (with optical density of ACPA below 20). Table 1 summarizes the descriptive characteristics of the subjects in the current Dataset 2 compared to Dataset 1.

### 2.2. Improvement in MCC-Seq Coverage

Using the definition of at least one aligned read in at least two samples, Dataset 1 had aligned reads to approximately 5.0 million CpGs on the autosome reference panel. In contrast, Dataset 2 covered slightly more CpGs in the genome (5,307,142 autosomal CpGs with at least one aligned read in at least two samples), and the number of overlapping sites included a high proportion of these (Table 2). Among Dataset 1’s captured sites (5,041,032 CpGs), 3,948,157 (78.3%) were shared with Dataset 2. After additional quality control steps to filter for sites with good coverage (see Methods Section 5.3 for details of QC), 1,305,080 CpGs were retained for parallel analysis in Dataset 1, and 4,259,820 in Dataset 2.

**Figure 1:**
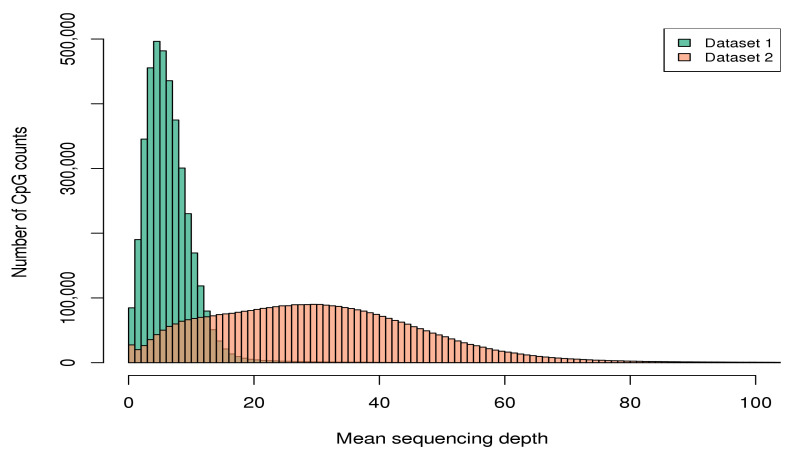
Histograms of mean sequencing depth across the CpGs covered by both datasets in the targeted region.

Dataset 2 shows not only a better consistency of coverage across samples, but also a substantial increase in average sequencing depth across CpGs in the targeted regions (i.e., 7× vs. 27× in Dataset 1 and Dataset 2, respectively). For example, among the 3,948,157 CpGs covered by both datasets in Table 2, 71,402 (1.8%) displayed median sequencing coverage of zero in Dataset 1 (i.e., at least half of the subjects had no reads at these sites). Moreover, 3,393,886 (86.0%) showed mean sequencing depth across samples smaller than 10× in Dataset 1. In contrast, in Dataset 2, 43,995 of these overlapping sites (1.1%) exhibited null median sequencing coverage, and merely 448,524 (11.4%) had mean sequencing depth smaller than 10×. Figure 1 compares the distributions of mean sequencing depth across the 3,948,157 overlapping CpGs.

### 2.3. Epigenome-Wide Association Studies (EWAS)

In this section and Section 2.4, we present the association results for Dataset 2, to showcase the full set of findings obtained from these high quality data. Comparisons with the results from Dataset 1 are shown in Section 2.5. A series of analyses were undertaken with different phenotypes and covariates, and Table 3 summarizes all the models fitted, the number of CpGs tested, and the number of identified DMCs and differentially methylated regions (DMRs). Detailed results of DMCs identified by each model was provided in Supplementary Tables S1–S6.

**Figure 2:**
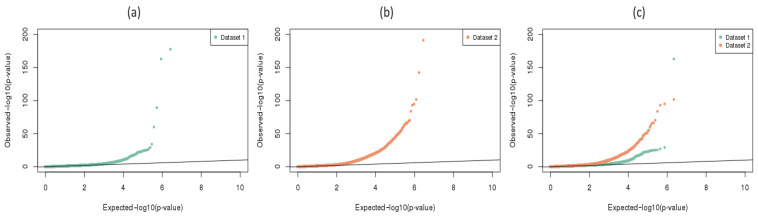
P-values QQ-plot of genome-wide ACPA-methylation associations. (**a**): 1,305,080 CpGs with good coverage in Dataset 1. (**b**): 4,259,820 CpGs with good coverage in Dataset 2. (**c**): 1,095,002 CpGs with good coverage in both datasets.

First we evaluated the difference in methylation levels between the 50 ACPA-positive and 54 ACPA-negative samples, using weighted binomial regression models that adjusted for age, sex, current smoking status and the first principal component of cell-type composition (Model I in Table 3). Among the 4,259,820 analyzed CpGs in Dataset 2, 19,472 ACPA-associated DMCs were identified at a false discovery rate (FDR) *q* value < 0.01 (detailed methods can be found in Section 5.4.1). Figure 2, panel (b), presents a QQ-plot for all tested CpGs in Dataset 2, and clearly indicates a substantial departure from the null hypothesis of no association. Of the 19,472 DMCs, 8581 showed higher methylation levels in the ACPA-positive subjects (hypermethylation), and 10,891 showed the opposite (hypomethylation).

The located DMCs then were grouped into small regions as DMRs, by combining at least three adjacent DMCs with the same direction of methylation changes and lying within 200 bp from each other (for our precise definition of a DMR, see Section 5.4.2). Of the 19,472 ACPA-associated DMCs addressed by Model I, 7028 (36.1%) could be clustered into 814 DMRs, among them 334 were hypermethylated regions and 480 were hypomethylated. The largest region included 86 hypomethylated DMCs on chromosome 2:39,470,818–39,471,848. However, most of the DMR sizes were relatively small: 280 DMRs (34.4%) included only 3 DMCs, and 381 DMRs (46.8%) contained 4–10 DMCs. It should be noted that our definition of a DMR was restrictive, in that all sites in the window had to pass the individual DMC significance threshold. Therefore, by our criterion, a large region could easily be broken into several sub-regions, or even fail to constitute a complete region, if it contains a few sites with poor coverage and hence insufficient power to detect associations.

In order to parallel analyses undertaken by the initial study [9], we also compared the methylation levels of eight study participants with self-reported RA to the other 104 non-RA subjects, regardless of their ACPA levels. Although there is evidence supporting that ACPA-positive and ACPA-negative RA have distinct etiology, response to treatment and severity [12], the numbers of participants with RA was too small in our study for subgroup analyses. As shown in Model V in Table 3, 18,874 RA-associated DMCs with *q* value < 0.01 were identified, of which 10,909 were hypermethylated and 7965 were hypomethylated. By grouping adjacent DMCs together, 843 RA-associated DMRs could be defined from 7403 DMCs.

**Figure 3:**
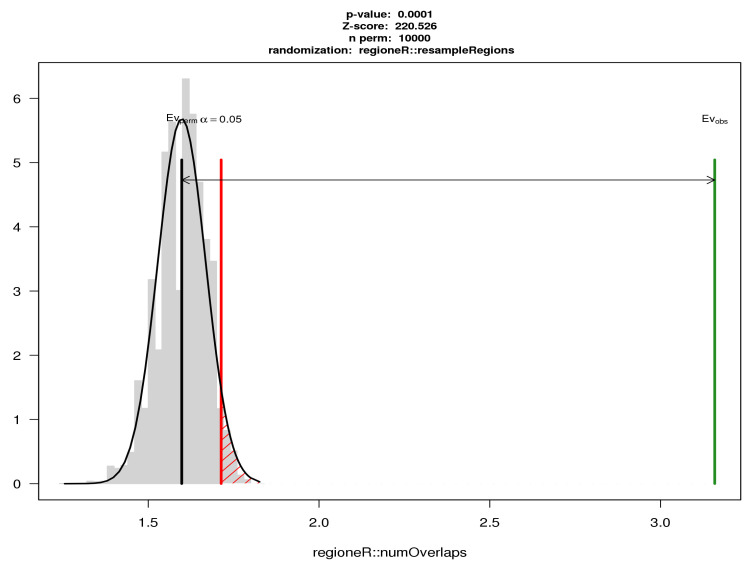
Numbers of sign-consistent overlapping DMCs between Model I and Model V generated by permutation analysis using regioneR. The X-axis represents the number of overlapping sites on a log10 scale, and the Y-axis specifies the probability density so that the histogram has a total area of one. The black curve on the left side shows the estimated null distribution, from 10,000 permutations, for the number of overlapping sites when significant DMCs for ACPA and RA are randomly selected from the 2,106,243 CpGs tested in both analyses and showing same direction of effect. The red vertical line denotes the 0.05 significance threshold for *p*-values. The green line on the right side shows the observed number of overlapping DMCs (1441) with same direction of effect, which is much larger than any of the 10,000 permutations.

We were interested in the cytosines and regions showing simultaneous relevance to the ACPA positivity and RA occurrence, since they may suggest epigenetic regulation pathways by which altered ACPA levels lead to an increased risk of RA. When matching the 18,874 RA-associated DMCs to the 19,472 ACPA-associated ones, we found 3302 sites overlapping by position, and 1441 of them showing the same direction of effect (i.e., either hypermethylated or hypomethylated in both ACPA and RA groups). Of these 1441 DMCs, more than one forth (392, 27.2%) fell into 43 DMRs. To confirm whether these overlaps were likely to have occurred by chance, a permutation-based method was used to estimate the null distribution of the number of overlapping sites (see method details in Section 5.5). It is clear from Figure 3 that the observed number of overlaps was far more than would be expected by chance.

### 2.4. Adjusting for Genetic Variants

In Section 2.3, methylation at tens of thousands of CpGs demonstrated an association with ACPA status. In fact, it could be argued that more associations were found than are plausible, given the power of this study (with sample size n= 104) and what is currently known about the biology of ACPA regulation. We have recently studied the impact of extra-binomial dispersion on tests of association using bisulfite sequencing DNA methylation data, and spurious significance can be seen when a sequence of methylation proportions do not follow the expected dispersion from a standard binomial distribution [13]. It has been implicated that some genetic variants, such as single nucleotide polymorphisms (SNPs), have a strong influence on the methylation levels of proximal CpGs [14,15], which may lead to over-dispersion of methylation proportions at these sites. Therefore, in Dataset 2, we identified cis-SNPs associated with methylation levels (cis-regulatory methylation quantitative trait loci, cis-meQTLs), and then included part of these cis-meQTLs in a second set of models testing for genetically adjusted association between methylation and ACPA status. These analyses aim to achieve three goals: (1) they may reduce over-dispersion, thereby leading to a better null distribution of *p*-values; (2) as will be discussed later, they reduce the inconsistency between the conclusions derived from Dataset 1 and Dataset 2; and (3) they allow us to ask the question of whether there may be more complex models relating SNPs, ACPA and methylation together. Given the very small number of individuals reporting RA diagnoses in our study, this kind of genetic adjustment was only undertaken for the outcome of ACPA positivity (not RA diagnosis).

#### 2.4.1. Identification of Cis-Meqtls

A series of local meQTL analyses were performed to identify SNPs with cis-effects (see Section 5.4.3 for details), using 108 participants from Dataset 2 where both SNP data and cell-type composition measurements were available. These analyses included 4,259,820 CpGs (i.e., all CpGs tested for ACPA associations in Dataset 2) and 4,742,491 SNPs (with minor allele frequency, MAF > 0.05 and imputed info score > 0.4). Only SNPs within the distance of 250 kbp from the closest CpG were tested for cis-effects, leading to analyses of 3,761,124,736 CpG-SNP pairs. At a *q* value threshold < 0.01, 17,609,708 CpG-SNP pairs were found to have significant associations; these pairs covered 330,721 CpGs and 2,663,706 SNPs. Among the 19,472 ACPA-associated DMCs identified in Model I for Dataset 2, there were 13,446 CpGs, where at least one cis-meQTL was found to be associated with the methylation. In total, 646,362 cis-meQTLs were located for these 13,446 DMCs, and no SNPs with cis-effects were identified for the remaining 6026 DMCs.

#### 2.4.2. Genetically Adjusted ACPA-Associated DMCs/DMRs

Our meQTL analysis identified many local SNPs that were associated with methylation at the same CpG, and in strong linkage disequilibrium with each other. To identify non-redundant SNPs for inclusion in genetically adjusted ACPA-methylation association models, adaptive lasso [16] was applied at each of the 13,446 ACPA-associated DMCs for a further variable selection, where the coefficients of all nearby cis-meQTLs in each DMC’s model were subjected to a $l_{1}$ penalty. The penalization parameters were chosen by five-fold cross validation with one standard error criterion (see Section 5.4.3 for more details of the adaptive lasso implementation). For each DMC, the subgroup of cis-meQTLs selected by adaptive lasso was then added into a relaxed ACPA-methylation association model, which adjusted for these genetic variants as well as the previous set of covariates (Model III in Table 3). Since there were 6026 DMCs without any identified nearby cis-meQLTs, no refitting was needed for them over the results from Model I in Table 3. The ACPA-associated DMCs with genetically adjusted *p*-values smaller than $3\times 10^{-5}$ (see Section 5.4.3 for the choice of threshold) were designated genetically adjusted DMCs (gDMCs). Similarly, we defined a genetically adjusted DMR (gDMR) by grouping three or more adjacent gDMCs.

As expected, many of the ACPA-methylation associations lost their strength after accounting for the cis-effects from meQTLs (Table 3). Among the 19,472 ACPA-associated DMCs (where 13,446 were refitted), 6314 (32.4%) retained significant as gDMCs, of which 1191 were from the refitted group. Of the 6314 identified gDMCs, 2415 showed hypermethylation and 3899 showed hypomethylation; 3167 gDMCs could be clustered into 302 gDMRs according to the direction of methylation changes. Another striking feature of these results is that, compared to the models without genetic adjustments, AIC and BIC decreased substantially by 50.8% and 49.9% on average, respectively, implying that variability in methylation levels were better explained than in models without genetic adjustments.

### 2.5. Agreement across Two Datasets

Although the collection of Dataset 2 was originally planned to replicate the results published in [9], the library coverage and read depth in Dataset 2 were different enough that parallel analyses were warranted. The 121 subjects in Dataset 1 for the parallel study are also described in Table 1 (see Section 5.1 for details of subject selection). Given the lower read depth in this dataset, when filtering CpG sites to keep only those with good coverage for analysis, a less stringent threshold was used: we required only 10 reads or more in at least 30 individuals, instead of 15 reads or more as was used for Dataset 2. Finally, there were 1,305,080 CpGs that met this criterion and were tested for association.

Models II, IV and VI in Table 3 summarize the parallel analyses undertaken for Dataset 1. All models adjusted for age, sex, current smoking status and the first principal component of cell-type composition, consistent with the analyses for Dataset 2. Model II, comparing ACPA-positive and negative individuals, found 853 ACPA-associated DMCs at *q* values < 0.01, of which 157 overlapped with the 19,472 ACPA-associated DMCs identified in Dataset 2. Panel (a) in Figure 2 shows the QQ-plot of association *p*-values from Model II for all tested CpGs in Dataset 1, and panel (c) compares the *p*-value QQ-plots from Model I and II for those CpG sites covered by both datasets.

**Figure 4:**
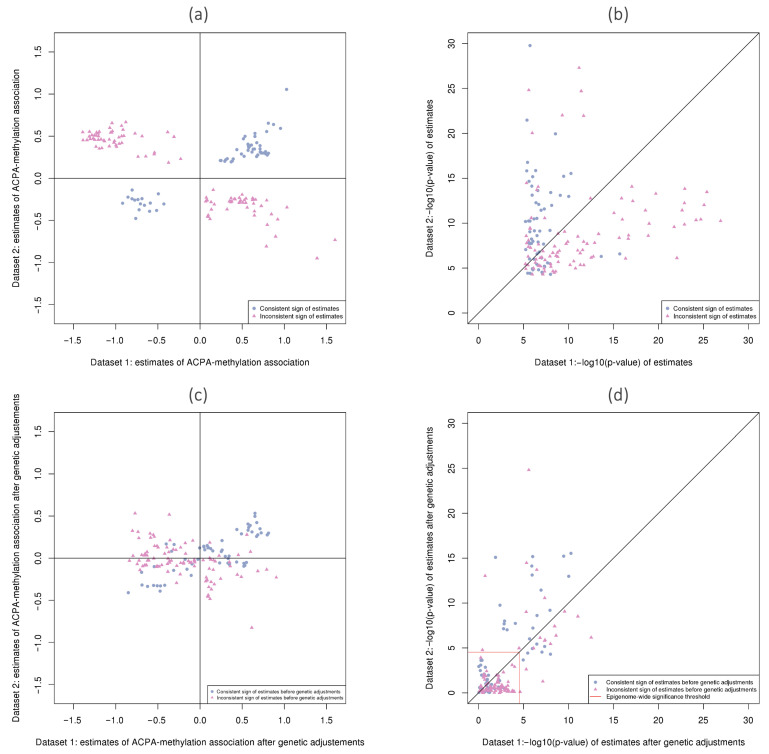
Scatter plots illustrating agreement between results for the 157 CpGs identified as demonstrating ACPA-methylation associations in both Dataset 1 and Dataset 2. (**a**) Estimated model coefficients (log odds ratios) from the EWAS binomial regressions. Points are colored in blue if the coefficient signs agree, and in pink otherwise; (**b**) −log10(*p*-values) from EWAS binomial regressions; (**c**) estimated coefficients (log odds ratios) after genetic adjustments; (**d**) −log10(*p*-values) after genetic adjustments including lines indicating significance threshold ($3\times 10^{-5}$). Shapes and colors for points in (**b**–**d**) correspond to those assigned in panel (**a**) for intuitive tracking of their changes.

For the 157 overlapping CpGs showing significant associations in both datasets at q<0.01, panel (a) in Figure 4 presents the estimated log odds ratios for the binomial model coefficients associated with ACPA positivity, and panel (b) is a scatter plot of the corresponding −log10(*p*-values). The matching shapes and colors of these points indicate whether the sign of the log odds ratio coefficients agreed between the two datasets as in panel (a). In fact, panel (a) in Figure 4 indicates that there were more DMCs where the direction of association differed between datasets (98 CpGs) than the DMCs that agreed in signs (59 CpGs). In panel (b), it can be seen that the vast majority of points corresponding to CpGs where the direction of effect is consistent between datasets (blue dots) display smaller *p*-values in Dataset 2. This would be in accord with larger amount of information in the newer dataset, as measured with higher read depth.

After accounting for the cis-regulatory genetic effects on methylation (model IV), 515 out of 853 ACPA-associated DMCs retained their significance as gDMCs. Of these, there were only 31 that overlapped with gDMCs identified in Dataset 2, and only 15 where the direction of effect was consistent between two datasets (including 14 hypermethylated and 1 hypomethylated). In Figure 4, panels (c) and (d) show coefficients and *p*-values after adjusting for nearby meQTLs, for the 157 ACPA-associated DMCs found in both datasets. In panel (c), it can be seen that after adjustment, there was a group of CpGs where the estimated log odds ratios were large and similar in magnitude (near 0.5 in absolute value) in both datasets. Panel (d) confirms that many of the inconsistent associations (pink triangles) may have been spurious associations, since most pink triangles have become non-significant after adjusting for genetic covariates. In fact, in (d), 110 out of 157 points now show *p*-values below the significance threshold, leaving only 47 sites significant in either dataset (and 31 in both). After taking the genetic contributions to methylation levels into account, there was more agreement between datasets among the remaining ACPA-methylation associations. Some of the CpGs that showed inconsistent directions in panel (a) now agree in sign in panel (c). Inconsistencies in conclusions were reduced or even eliminated by adjusting for the nearby genetic variants.

**Figure 5:**
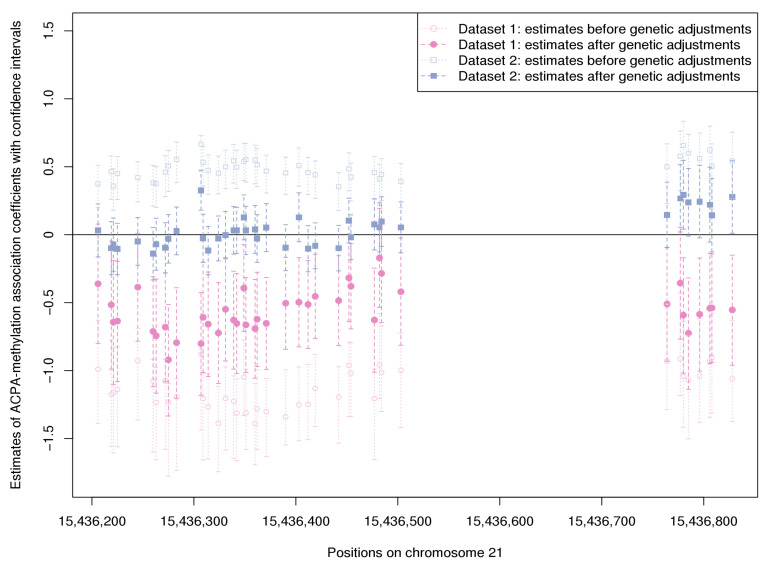
Estimated coefficients and confidence intervals for association between methylation and ACPA status from binomial regressions in Dataset 1 and Dataset 2, with and without inclusion of meQTL covariates.

In order to better visualize how ACPA-methylation associations may be influenced by genetic variation, Figure 5 shows the log odds ratio estimates of association with ACPA status, and their confidence intervals, for two adjacent DMRs on chromosome 21 that were well captured in both datasets. The estimates obtained from both datasets are shown, before and after including cis-SNP variables into the models. When no SNPs were included as covariates, the sign of coefficients for ACPA disagreed between Dataset 1 and 2, but both met our threshold for statistical significance. That is, the DMCs showed significantly higher methylation levels among the ACPA-positive individuals in Dataset 2, but significantly lower methylation levels in Dataset 1. However, after accounting for the genetic effects from 5 SNPs (selected from 1438 potential meQTLs by adaptive lasso), the results of two datasets became less divergent, and the coefficient estimates became closer to zero. Most of the positive associations in Dataset 2 lost strength after adjusting for genetic effects, but in Dataset 1 they remained statistically significant below zero.

**Figure 6:**
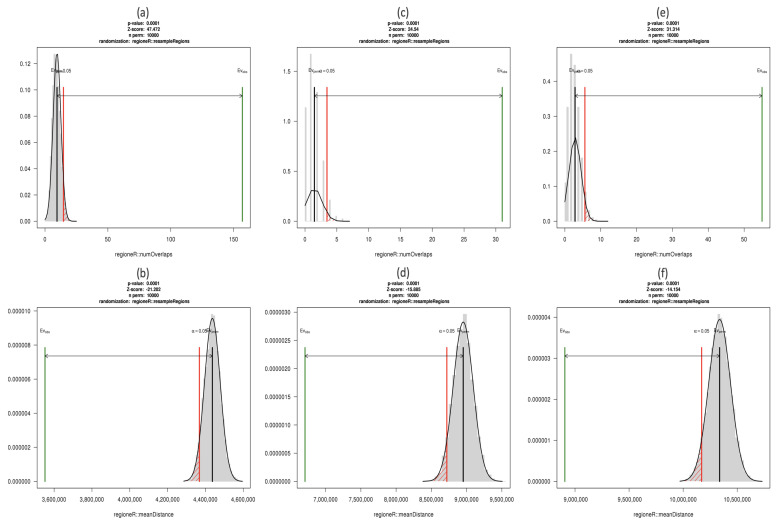
Null distributions and corresponding 0.05 *p*-value thresholds for overlap or agreement, from 10,000 permutations in different cases: (**a**) the number of overlapping ACPA-associated DMCs between two datasets; (**b**) the mean distance between ACPA-associated DMCs in two datasets; (**c**) the number of overlapping ACPA-associated gDMCs between two datasets; (**d**) the mean distance between ACPA-associated gDMCs in two datasets; (**e**) the number of overlapping RA-associated DMCs between two datasets; (**f**) the mean distance between RA-associated DMCs in two datasets. The X-axis represents the number of overlapping sites in (**a**,**c**,**e**) and the mean distance in (**b**,**d**,**f**), the Y-axis specifies the density so that the histogram has a total area of one. The green vertical bars represent what we actually observed in each case. The sampling universe for permutations was CpGs tested in both datasets.

We also performed a parallel analysis of Dataset 1 comparing participants with self-reported RA to all others. Model VI analyzed 1,295,623 CpGs in Dataset 1 (Table 3) and identified 258 RA-associated DMCs, of which 55 overlapped with the results of Model V (Dataset 2). Given the large number of CpGs analyzed, we asked whether the DMCs found in Dataset 2 overlapped with the DMCs in Dataset 1 more than expected by chance. Figure 6 shows results of random permutation analyses to answer this question, for ACPA-associated DMCs, ACPA-associated gDMCs and RA-associated DMCs, respectively. Similar to Figure 3, the three plots in the first row compare the null distribution of the number of overlapping CpGs obtained from random permutations with the observed number of overlaps. Clearly, many more overlapping sites were found than would be expected by chance.

However, the actual number of overlapping sites (regardless of permutation test results) was smaller than had been hoped, due to the changes in library design and coverage. To reduce the impact of such technical design features when assessing replication of findings, we asked whether the DMCs in Dataset 2 were significantly closer to the DMCs in Dataset 1. We computed the mean of the distances from every DMC in Dataset 2 to the closest DMC in Dataset 1. A significantly closer distance implies that the DMCs are likely to have been identified from a nearby differentially methylated region, even though only a small fraction of CpGs overlap. In Figure 6, the three plots in the second row compare the null distribution of the mean distances with the observed mean distances, and show that there was a clear tendency for the DMCs from the two datasets to be spatially close.

### 2.6. Replication of Results Found in the Initial Study

Although it is more meaningful to compare between the results from two datasets that adopted the same QC criteria and model constructions as we did in the previous section, it is still worthwhile to directly compare our findings based on the new dataset with the published results using Dataset 1 [9]. In the original study, 1909 ACPA-associated and 955 RA-associated DMCs were identified from 4,635,909 and 4,109,916 CpGs in Dataset 1, respectively. Among them 1783/1909 ACPA-associated and 872/955 RA-associated sites were contained in the test sets of Dataset 2 and reanalyzed in our study. By comparing the results of model I and model V with those of the corresponding models in the previous study, 410 ACPA-associated DMCs and 156 RA-associated DMCs were replicated in our research. Among these overlapping sites, 230/410 ACPA-associated DMCs and 110/156 RA-associated DMCs were reported to have consistent methylation status (hypermethylated or hypomethylated) when comparing the two studies. By grouping these consistent DMCs together, 11 consistent ACPA-associated DMRs and 6 consistent RA-associated DMRs could be constructed from 187 and 91 corresponding DMCs, respectively. Table 4 summarizes the DMCs and DMRs identified in both studies, and more details of these co-localized cytosines are provided in Supplementary Tables S7 and S8.

## 3. Integrating EWAS Findings with GWAS Catalog Loci

In the previous section, we investigated DMCs and DMRs associated with ACPA positivity and RA, and validated hundreds of loci demonstrating differential methylation levels. In our meQTL study, we detected local correlations between SNPs and differential methylation for part of our identified ACPA-associated DMCs, showing that genotypes at specific loci can result in different patterns of DNA methylation. A complementary lens for situating our methylation-phenotype associations in a broader context is to look at the co-localization of our identified DMRs and loci reported in published GWAS for ACPA positivity and RA, as DNA methylation can be an important molecular-level mediator that links complex disease traits and genotypes [7].

By linking loci to genes, here we look at overlaps between genes highlighted as markers of ACPA positivity/RA from both the GWAS Catalog and our epigenetic analyses. ACPA/RA-associated DMRs and SNPs that mapped to the same protein-coding genes may support mediation of SNP-phenotype associations through DNA methylation, where differences in cases and non-cases may directly lead to expression of proteins that are part of the pathophysiology of ACPA positivity/RA. To ensure reliability as well as reproducibility of our results, only the ACPA and RA-relevant DMRs concluded from Dataset 2 were considered in these explorations.

### Integration with Published GWAS Knowledge

Published SNPs associated with RA disease at genome-wide significance $5\times 10^{-8}$ were extracted from the GWAS Catalog (https://www.ebi.ac.uk/gwas (accessed on 31 March 2021)). Download information and filtering strategies are described in Section 5.6.1. The resulting list contained 585 SNPs, and of these, 435 had already been mapped to 295 unique protein-coding genes.

Then, DMRs highlighted in Section 2.3 for association with ACPA positivity or RA were also mapped to the nearest protein-coding gene. Specifically, we assigned a DMR to a gene when it overlapped the 1–5 kbp upstream of TSS, the promoter or the gene body. For interpretability, SNPs and DMRs that mapped to intergenic regions or to long non-coding genes were not included for subsequent analyses. Of our 814 ACPA-associated DMRs, 410 mapped to the TSS upstream or gene body of 403 unique protein-coding genes. When comparing these genes to those associated with the GWAS Catalog loci, there were 14 genes sharing between two sets. Similarly, for our 843 RA-associated DMRs, 398 of them could be mapped to 376 protein-coding genes, and of these, 7 overlapped with those in the GWAS gene set. Table 5 summarizes the number of assigned genes and the symbols of those captured both in the GWAS Catalog and by our EWAS. Notably, the genes *ERICH1*, *ZNF595*, *SPAG1*, and *TP73* were contained in all sets as shown in Table 5. The genes *HLA-DRB1* and *HLA-DRB5*, well-known to be associated with RA risk, were identified in both the GWAS set and ACPA-DMR set.

We then examined which biological functions relevant to RA pathogenesis were associated with our gene sets. We performed Gene Ontology analyses on each of the three gene sets (generated from DMRs for ACPA positivity, DMRs for RA, and GWAS Catalog SNPs for RA), to test whether these genes were significantly enriched in some molecular functions or biological processes. The three gene sets were separately used as inputs to the R package gprofiler2 [17] for functional profiling. For details of the parameter settings, see Section 5.6.3.

**Figure 7:**
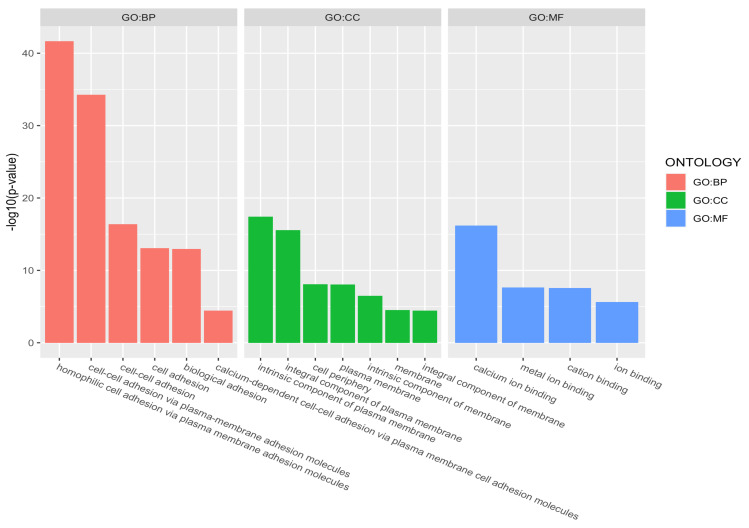
Gene Ontology terms showing over-representation with corrected *p*-values <0.05, for the list of genes derived from the ACPA-associated DMRs. Abbreviations: BP: Biological Process; CC: Cellular Component; MF: Molecular Function.

Figure 7 shows GO terms with significant over-representation in the gene set for ACPA-associated DMRs, at an adjusted *p*-value <0.05. Complete GO annotation results for all three sets are provided in Supplementary Tables S9–S11. Significantly, there was evidence for enrichment of four GO terms in all sets; Table 6 lists the IDs and names of these four terms with corresponding enrichment *p*-values. In fact, three biological process (BP) terms belong to the same hierarchy. Specifically, cell–cell adhesion (GO:0098609) is a subtype of cell adhesion (GO:0007155), and cell adhesion is a subtype of biological adhesion (GO:0022610).

We were particularly interested in cell–cell adhesion (GO:0098609), considering the fact that ‘child’ terms are more specialized than their ‘parent’ terms. In the gene set associated with the RA GWAS SNPs, 43 genes mapped from 86 risk variants were involved in cell–cell adhesion; the other two gene sets derived from ACPA-associated and RA-associated DMRs had 67 and 58 genes involved in this specific biological process, annotated from 55 and 49 corresponding DMRs, respectively. Table 7 lists the genes involved in cell–cell adhesion for each of the profiled gene sets. *HLA-DRB1* and *KIF26B* were presented in the GWAS set and ACPA-DMR set at the same time; *AP3D1*, *CDHR3*, *MAD1L1*, *MAG*, *MBP*, *PDGFRA*, *PKP3*, *SCRIB*, *SDK1*, *UBASH3B*, and a group of clustered protocadherin (*PCDH*) genes were shared between the ACPA-DMR and RA-DMR sets.

**Figure 8:**
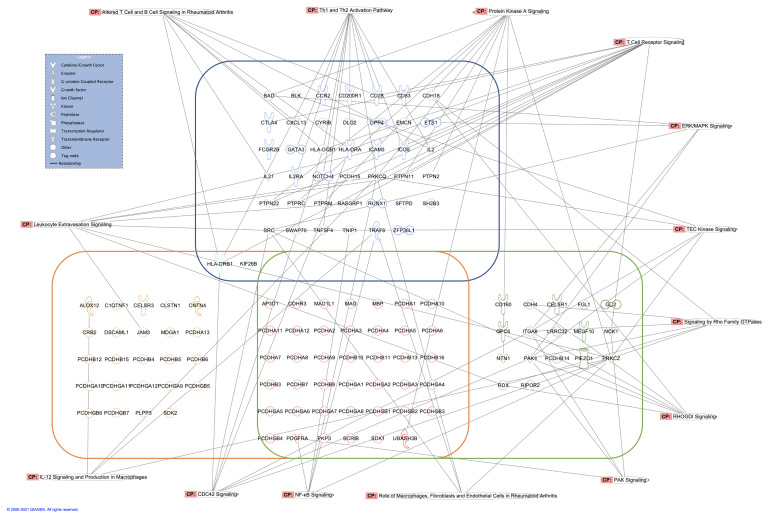
Overlaid IPA canonical pathways for the gene sets involved in the biological process of cell–cell adhesion (GO:0098609). Genes for RA GWAS SNPs, ACPA-associated DMRs and RA-associated DMRs are surrounded by the blue, orange and green boxes, respectively. The genes shared across sets are placed in the intersection areas. The genes in highlighted canonical pathways are connected to the pathway names by lines.

In order to gain a more systematic understanding of how cell–cell adhesion may play a role in RA pathogenesis, and how the differentially methylated genes and SNP-associated genes involved in cell–cell adhesion may communicate, the three groups of genes in Table 7 were overlaid with the curated canonical pathways from the database of QIAGEN Ingenuity Pathway Analysis (IPA) software. In Figure 8, the three groups of cell–cell adhesion genes are surrounded by different boxes, and the genes shared across groups are placed in the intersection areas. Several top signaling pathways related to immunoreaction, such as NF-κB signaling, Th1 and Th2 activation, leukocyte extravasation signaling and pathways of migration-related proteins, such as Rho family of GTPases, are highlighted around the genes.

## 4. Discussion

In this manuscript, we have identified many novel DMCs and DMRs associated with ACPA positivity and RA diagnosis. Our parallel studies have validated hundreds of cytosines with consistent status of methylation, and their overlaps were confirmed by a series of permutation analyses. Furthermore, we have identified and validated some ACPA-associated gDMCs by adjusting for cis-regulatory genetic effects, showing that genotypes at specific loci can result in different patterns of DNA methylation. Finally, we have placed a subset of our findings into context by looking at the DMRs that overlap with RA-associated loci in the international GWAS Catalog at gene level, the protein-coding genes associated with these DMRs are enriched in the biological process of cell–cell adhesion and involved in multiple immune-related pathways.

Dataset 2 was originally planned as a validation of results obtained in Dataset 1. However, given the increased resolution and quality of Dataset 2, we have presented results in parallel. If we had only focused on the overlapping sites with high sequencing coverage in both datasets, we would have missed many interesting associations. Therefore, further validation, possibly with a genome-wide approach, merits consideration.

Our results highlight many protein-coding genes as potentially involved in ACPA positivity and RA-relevant differential methylation. In the original study using Dataset 1 [9], 475 unique protein-coding genes were reported to be associated with DMRs from ACPA ordinal regression analysis (Supplementary Table S4 of the original study). Comparing with our results obtained from Dataset 2, 94 genes were replicated, in that ACPA-associated DMRs were identified in their functional regions (from 1–5 kbp upstream of TSS to gene body). Moreover, RA-associated DMRs were simultaneously identified in the functional regions of 44/94 genes, including *ERICH1*, *SPAG1*, *ZNF718*, *AHRR*, *EEPD1*, *ARHGAP39*, *TBCD*, *HIVEP3*, *CSGALNACT1*, *CCDC144A*, *SLC6A18*, *KCNN3*, *SDK1*, *CDHR3*, *MYOM2*, *STK32C*, *LRRC27*, *LSP1*, *CBFA2T3*, *RPH3AL*, *LDLRAD4*, *FCER2*, *MYO18B* and a group of genes from the clustered protocadherin family. In addition, the differentially methylated genes involved in immune-related pathways in the original study included *CARD11*, *CSF2*, *FCGR2A*, *IFNAR2*, *MAP3K7*, *MAPK9*, *NFATC1*, *NFKBIA*, *PAK4* and *SOCS3*. Most of them, except for *CSF2*, *FCGR2A*, *MAPK9* and *SOCS3* were replicated as also associated with either of ACPA/RA-relevant differential methylation in the second dataset.

Among all protein-coding genes annotated from RA GWAS loci, we have identified both ACPA-associated and RA-associated DMRs in the functional regions of four genes—*ERICH1*, *SPAG1*, *ZNF595*, and *TP73*. Among them, *ERICH1*, encoding the glutamate rich 1 protein, has two hypermethylated RA-associated DMRs and one hypermethylated ACPA-associated DMR in its gene body. Glutamate was found to participate in adhesion, migration, proliferation, survival and activation of T cells [18]. Molecular biological analyses discussed the role of glutamate in synovial fibroblasts and possible involvement of glutamatergic signaling in the pathophysiology of RA [19,20]. A complete list of identified DMRs in the regions of RA GWAS genes can be extracted from Supplementary Tables S1 and S5.

Our Gene Ontology analyses have detected groups of genes that show significant enrichment for the biological process of cell–cell adhesion. Cell adhesion molecules (CAMs) are expressed on the membrane of cells and enable cell–cell interactions; they form a molecular backbone of a series of important physiological and pathological processes such as immune and inflammatory response. The expression of intercellular adhesion molecule (ICAM)-1 and vascular cell adhesion molecule (VCAM)-1—two members of the immunoglobulin superfamily—have been found to be markedly upregulated in the synovial membrane of RA patients [21,22,23]. Kurowska et al. [24] discussed the role of ACPA in the early phases of RA indicating that the presence of ACPA is highly associated with the activation of synovial fibroblasts, which upregulate CAMs and secrete proinflammatory cytokines in RA patients.

Among the genes involved in cell–cell adhesion as classified by gene ontology analysis, we focused more on those associated with DMRs identified through our EWAS, particularly two genes also reported to be affected by RA GWAS loci. One is *HLA-DRB1*, which belongs to the class II of the gene family major histocompatibility complex. The molecules encoded by *HLA-DRB1* are initially expressed on the cell surface of immune cells such as B cells and activated T cells, and the *HLA-DRB1* alleles are well known to be the largest genetic risk factors of RA [25]. In EWAS, we detected a hypermethylated ACPA-associated DMR (chr6:32,551,741-32,552,477) comprising 11 DMCs that covered the promoter region and gene body of *HLA-DRB1*. Nevertheless, our meQTL analysis revealed that the DMCs in this DMR were strongly affected by cis genetic effects, and the ACPA-DMC associations disappeared after genetic adjustments. These results are consistent with those of Liu et al. (2013) who found that the DNA methylation alterations in this region mediate genetic risks for RA [7]. The other gene is *KIF26B*. Chen et al. (2017) analyzed differential gene expression between osteoblasts from RA patients and controls. They identified upregulation of *KIF26B* in RA osteoblasts, and their pathway analysis suggested that *KIF26B* was involved in diseases and functions related to hereditary disorders, immunological disease, and organismal injury [26].

We noticed there were 47 clustered protocadherin (*PCDH*) genes involved in cell–cell adhesion, associated with 28 DMRs identified in our ACPA analyses, of which 25 regions were hypomethylated and three regions were hypermethylated. There were 31 *PCDH* genes where we also identified 18 RA-associated DMRs, of which 17 regions were hypomethylated and one region was hypermethylated. Among these DMRs identified in the *PCDH* region, eight RA-associated DMRs overlapped with eight ACPA-associated DMRs, and they all showed hypomethylation in both ACPA and RA groups. *PCDH*s are a group of cell–cell adhesion proteins that belong to the cadherin family [27]. Gomez et al. (2016) analyzed blood samples from monozygotic twins who were discordant for ACPA-positive RA, and they identified several DMRs associated with *PCDHB5* and *PCDHB14* [28]. Their results are consistent with ours, since we detected a hypomethylated RA-associated DMR in the exon of *PCDHB14* and two hypomethylated ACPA-associated DMRs in the exon of *PCDHB5*. In addition, Hass et al. (2006) conducted a cDNA microarray analysis in monozygotic twins to identify RA-specific gene expression profiles, and they found *PCDHB16* had significantly higher mRNA expression in twins with RA compared with their healthy cotwins [29]. In our study, we identified three hypomethylated ACPA-associated DMRs and one hypomethylated RA-associated DMR in the promoter of *PCDHB16*, and also one hypomethylated ACPA-associated DMR in the exon of *PCDHB16*. The *PCDH* gene clusters (tandem-arrayed *PCDHA*, *PCDHB* and *PCDHG*) have a genomic organization similar to B-cell and T-cell receptor gene clusters [30], and their diversity relies on epigenetic regulation and alternative transcription [31]. Therefore, the increased DNA methylation variability detected in their functional regions could imply a role in self-recognition and autoimmunity. For instance, one study has shown that *PCDH18* protein was an activation marker of CD$8^{+}$ memory T cells [32]. We speculate that the prevalent hypomethylation in this area of the genome may up-regulate the expression of *PCDH* cell adhesion proteins, proposing epigenetic connection in the progression from ACPA-positivity to clinical RA.

The IPA software for the canonical pathway reference highlights multiple pathways and genes related to immune response and cell migration. For example, uncontrolled leukocyte extravasation is an important characteristic of chronic autoimmune diseases such as RA [33]. *JAM3*, the junctional adhesion protein that mediates heterotypic cell–cell interaction, plays a central role in leukocytes extravasation by working with other adhesion molecules and facilitating transmigration through the endothelium [34]. Arrate et al. (2001) provided evidence that *JAM3* is up-regulated following T cell activation, showing its potential contribution to pathologies with T cell involvement [35]. *RDX* has also been implicated in leukocyte extravasation signaling through its ability to influence cell communication. In endothelial cells, *RDX* protein along with other linking proteins offers attachment points on plasma membrane for organelles, which provide a route for communication with ICAM-1 and VCAM-1 and then controls leukocyte transmigration [36]. *PDGFRA*, encodes a cell surface receptor for platelet-derived growth factor which activates the nuclear factor (NF)-κB signaling pathway. The activation of NF-κB positively regulates cell adhesion molecule and plays a key role in the pathogenesis of RA [37]. Moreover, *PDGFRA* was identified and validated as a direct target of microRNA-146b in hematopoietic cells; recent work indicated that microRNA-146 family has a close relationship with inflammation and autoimmune diseases [38,39]. Apart from these, other pathways also show promise for the treatment of RA. For example, Tec family kinases have been demonstrated to play a key role in inflammation and bone destruction [40]; the Rho family GTPases members (RhoA, Cdc42 and Rac1) have been reported to be involved in the migration and invasion of RA fibroblast-like synoviocytes [41]; PAK signaling is known to activate JNK pathways that contribute to the induction of immune cell death [42]; mitogen-activated protein kinases/extracellular signal-regulated kinases (MAPK/ERK) signaling pathway is involved in proliferation and migration of RA fibroblast-like synoviocytes [43]. Of note, Chu et al. (2016) identified a non-canonical PI3K-Cdc42-PAK-MEK-ERK signaling pathway promoting apoptosis in human neutrophils, which are crucial players in the generation of the inflammatory response [44]. We noticed the differentially methylated gene *PRKCZ*, encoding for protein kinase C zeta, was involved in most pathways together with RA GWAS gene *PRKCQ*. *PRKCZ* has been found to be hypermethylated in RA fibroblast-like synoviocytes [45,46]. Besides, two pathways along with genes involved—IL-12 signaling and production in macrophages, role of macrophages/fibroblasts/endothelial cells in RA, might be bridged through this gene. These findings may provide important insights into the pathogenesis of RA and introduce potential therapeutic targets.

Some of our analytic choices could be debated. For example, when analyzing the RA-methylation associations, we decided to ignore patients’ ACPA status since there were so few RA subjects, and this was the same approach taken by [9] in their analysis of Dataset 1. However, RA without ACPA-positivity could be considered as a different etiologic process [47]. When performing the meQTL analyses, to use an existing computationally-efficient R package originally written for continuous gene expression data, we used the arcsine transformations [48] of methylation proportions so that ignored the read depth, this choice may have decreased power for meQTL detection. We also found several DMCs where the direction of association differed between Dataset 1 and Dataset 2. For many of these sites, a strong meQTL was found nearby. It is possible that the discrepancies in the methylation patterns represents Simpsons paradox, i.e., cases of very strong confounding by the SNP effects.

In summary, our integrative analysis shows that genetic and epigenetic modifications may be working together on the expression of a group of protein-coding genes that enriched in the biological function of cell adhesion. Our results point to these interesting candidate genes for ACPA positivity and RA, and highlight the importance of cell adhesion for chronic inflammatory disease such as RA.

## 5. Methods

### 5.1. CARTaGENE Subjects

**Figure 9:**
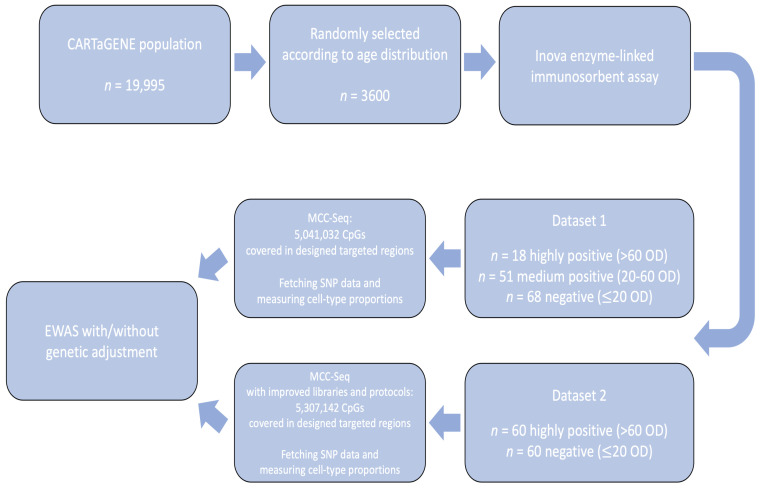
Procedures for data collection and analysis.

The CARTaGENE cohort [49] is a sample of approximately 40,000 individuals from four regions in Quebec, Canada, with demographic and health questionnaires as well as stored blood, serum and urine samples. Stored serum samples from 3600 participants among the first 19,995 recruited subjects were analyzed for ACPA levels with INOVA enzyme-linked immunosorbent assay (Quanta Lyte, CCP3 IgG: Inova Diagnostics Inc., San Diego, CA, USA) [9]. From these results, Shao et al. (2019) had selected 137 subjects with either high ACPA (optical density of ACPA > 20) or low ACPA (optical density of ACPA ≤ 20) levels to undergo custom methylC-capture sequencing (MCC-Seq) [10]. Here in this replication study, 120 additional individuals were selected with either highly positive (optical density of ACPA > 60) or negative (optical density of ACPA ≤ 20) ACPA levels, and their stored blood samples were sent for MCC-Seq. Blood cell composition and genotype information of the sequenced samples were measured or collected from different pipelines during sequencing. Figure 9 provides an overview of the data collection process in the initial and this replication study.

As we defined earlier, we refer to the 137 subjects analyzed in [9] as Dataset 1, and the 120 subjects in this replication study as Dataset 2. From Dataset 1, there were 15 participants whose cell-type composition data were not available, and 1 subject whose ACPA level was low-positive (OD = 29.67); these individuals were removed from parallel analysis to be consistent with our selection protocol for Dataset 2, leaving only medium-positive (40< OD ≤60) and high-positive (OD >60) subjects for the ACPA-positive group in Dataset 1.

### 5.2. Methylation Sequencing

The pooled samples underwent targeted custom-capture bisulfite sequencing (MCC-Seq) described in [10]. Compared to Dataset 1, the immune capture panel was developed from the first generation to the third generation for a more comprehensive profiling, and library preparation was changed from using the KAPA library kits to the Lucigen NxSeq AmpFREE Low DNA library kits, along with the matched protocols. Multiplex sequencing was performed in 16-plex from 63 ng DNA and 15-plex from 66 ng DNA for Dataset 1 and 2, respectively. Furthermore, in the targeted regions, the desired average read depth for Dataset 2 was close to 40× in contrast to 15× for Dataset 1.

### 5.3. Cleaning and Quality Control for MCC-Seq Data in Dataset 2

- Alignment: Stranded bisulfite treated sequences were aligned against reference genome hg19 using Novoalign, duplicate fragments were removed with samtools. Methylation status of aligned cytosines for each sample were called by Novomethyl. Details of alignment and data cleaning for Dataset 1 can be found in [9]; alignment was performed with Bismark and the methylation levels were calculated after combining forward and reverse strands.
- SNP calling and imputing: Genotyping data was fetched from CARTaGENE, the details of pipeline used for the quality control of CARTaGENE’s genotyping data can be found on their website (https://www.cartagene.qc.ca/en/researchers/catalogue/genetic-data (accessed on 31 March 2020)). Because the data was generated through 5 genotyping arrays: Axiom, Omni 2.5 M, GSA760, GSA4224, GSA5300, the genotyping data on the same samples were then imputed using the Sanger imputation service (https://www.sanger.ac.uk/tool/sanger-imputation-service/ (accessed on 7 April 2020)), and the guidelines listed on the website were followed.
- Filtering of CpG sites: Analyses were restricted to CpG sites on the autosomes. Any CpG site where all reads were either methylated or unmethylated across all samples (i.e., no variability exists) were eliminated from analyses. In Dataset 2, sites were retained for either ACPA or RA association studies if there were at least 30 samples (study participants) with read depth ≥15×. In re-analyses of Dataset 1, this read depth restriction was relaxed to ≥10× to reach a balance between quality and quantity.

### 5.4. Statistical Analysis

#### 5.4.1. Testing for Differential Methylation with ACPA and RA Status

Weighted binomial regression models were used to investigate site-wise DNA methylation differences in ACPA-positive vs. ACPA-negative subjects. The number of methylated reads for each sample (at each CpG site) was modelled as a binomially distributed response variable conditional on total read depth. The primary covariate of interest was the ACPA status or—either positive or negative. Additional covariates included in all models were age, sex, current smoking status, and the first principal component of five measured cell-type proportions (monocyte, lymphocyte, neutrophil, eosinophil, basophil). Due to the fairly small sample size in this study, model fits became unstable when covariates were included for all (but one) of the different cell type sub-components. Therefore, the information in the five cell-type compositions was transformed with a singular value decomposition, and the first component, which explained 92.1% of variance, was included in all our models.

The weighted binomial regression model for each site *k*, and for an individual *i* was formulated as:

(1) $$M_{i}^{k}∼Binom\left(M_{i}^{k}+U_{i}^{k},p_{i}^{k}\right)$$

where

(2) $$\mathrm{logit}\left(p_{i}^{k}\right)=\beta_{0}^{k}+\beta_{1}^{k}{\mathrm{ACPA}}_{i}+\beta_{2}^{k}{\mathrm{age}}_{i}+\beta_{3}^{k}{\mathrm{sex}}_{i}+\beta_{4}^{k}{\mathrm{smoking}}_{i}+\beta_{5}^{k}{\mathrm{PC}1}_{i}$$

where $p_{i}^{k}$ represents the expected mean methylation proportion for the *i*-th subject at CpG site *k*. This model was almost identical to the model used in the initial study [9], with one difference that we reduced multiple variables for cell-type proportions and used only the first principal component. All models were implemented based on the R function glm().

Significance was assessed by the false discovery rate [50] across the autosomes. The *q* values of the estimated coefficient of interest (${\hat{\beta }}_{1}$) were calculated by using R package qvalue [51] with default parameters. The CpGs where *q* value was less than 0.01 were considered to demonstrate association, and defined as differentially methylated cytosines (DMCs). The DMCs with positive coefficients for the ACPA covariate (i.e., ${\hat{\beta }}_{1}>0$) were referred to as hypermethylated DMCs, and those with negative coefficients (${\hat{\beta }}_{1}<0$) were termed hypomethylated DMCs.

Similar analyses were used to identify RA-associated DMCs across the autosomes. Specifically, individuals with self-reported RA were compared to all other study participants, and the following modified regression model was applied to each CpG site:

(3) $$\mathrm{logit}\left(p_{i}^{k}\right)=\beta_{0}^{k}+\beta_{1}^{k}{\mathrm{RA}}_{i}+\beta_{2}^{k}{\mathrm{age}}_{i}+\beta_{3}^{k}{\mathrm{sex}}_{i}+\beta_{4}^{k}{\mathrm{smoking}}_{i}+\beta_{5}^{k}{\mathrm{PC}1}_{i}$$

where ${\mathrm{RA}}_{i}$ indicates the RA status of *i*-th subject (1 = yes, 0 = no). The CpGs with *q* values less than 0.01 are called as RA-associated DMCs.

#### 5.4.2. Differentially Methylated Regions (DMRs)

**Figure 10:**
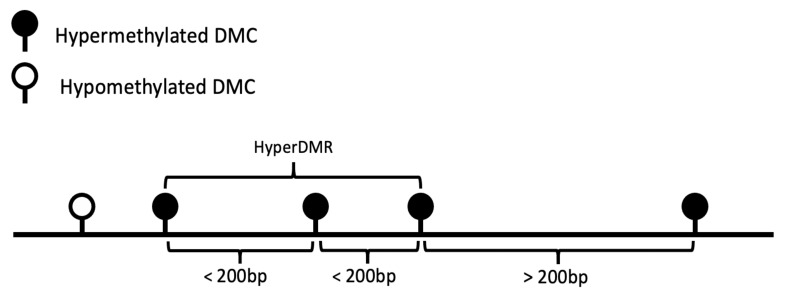
An example of a hypermethylated DMR.

DMRs were constructed by merging spatially adjacent hypermethylated/hypomethylated DMCs together. That is, two adjacent DMCs located within a distance of 200 bp are considered as contiguous, and three or more contiguous DMCs with the same sign of coefficient estimates ${\hat{\beta }}_{1}$ (hypermethylated or hypomethylated) were defined to constitute a DMR. Figure 10 gives an example of how a hypermethylated DMR was constructed from three consecutive DMCs.

#### 5.4.3. Genetic Effects on DNA Methylation and ACPA Status

In order to understand how SNPs may influence the associations between DNA methylation and ACPA status, a series of additional analyses were performed including cis-SNPs into the models. For CpG sites where differential methylation was observed, we examined whether the associations remained strong after adjusting for selected neighboring genetic variants.

Imputed SNPs with INFO score greater than 0.4 were considered as acceptable well-imputed variants. Taking the sample size into consideration, we retained only SNPs with minor allele frequency (MAF) greater than 0.05 for subsequent analyses. Multiallelic sites and multiple variants sharing the same basepair coordinate or allele codes were excluded.

For each CpG analyzed in the univariate association studies, SNPs located within 250 kbp upstream or downstream (i.e., a window of 500 kbp) were paired with the CpG and tested for cis-effects on methylation, this is often referred to as meQTL analysis. Fast meQTL analyses for all CpG-SNP pairs were implemented by the R package MatrixEQTL [52]. Since the package currently supports only linear models, the methylation proportion, as a dependent variable, was linearized by an arcsine transformation. This transformation was originally proposed by Yu (2009) [48] for variance stabilization of binomially distributed data. Similar to the DMC analysis, all meQTL models were adjusted for age, sex, current smoking status and the first principal component of the cell-type composition measurements. False discovery rate *q*-values were provided as output by the package, and SNPs with *q*-values less than 0.01 were considered to be cis-meQTLs. These SNPs often have strong effects on the methylation levels, causing the over-dispersion of methylation proportions, and may confound the DNA methylation–ACPA relationships.

To control for these genetic effects in the ACPA-methylation association analyses, we planned to re-analyze all the ACPA-associated DMCs with cis-meQTLs as additional covariates in the regression model (2). However, the meQTL analysis often identified a very large number of associated SNPs for each CpG, such that there would be more covariates than our sample size (p≫n) if they were all included. Furthermore, many of these associated cis-SNPs are in strong linkage disequilibrium. Therefore, we firstly reanalyzed each DMC using binomial regression with adaptive lasso including an $l_{1}$ penalty on the genetic terms [16,53]. The adaptive weights allow for different degrees of penalization for the various associated SNPs in the $l_{1}$ penalty. The penalty parameter was chosen by following the principle of one standard error from the minimum cross-validated error, using five-fold cross validation. After fitting the adaptive lasso, any cis-meQTLs that retained non-zero coefficients were kept for the following genetically adjusted analysis. These procedures were implemented using the R package glmnet [54]. Then, a series of non-penalized binomial regressions were refitted for those DMCs with at least one preserved cis-meQTL, incorporating the selected subset of SNPs as well as other environmental factors as covariates. After inclusion of genetic factors into models, previously identified ACPA-associated DMCs with adjusted association *p*-values less than an epigenome-wide significance threshold at $3\times 10^{-5}$ were determined as genetically adjusted DMCs (gDMCs). The threshold at $3\times 10^{-5}$ was roughly equivalent to 0.01 FDR among the universe of all 4,259,820 CpGs determined by converting from the *q*-value distribution back to the *p*-value distribution.

### 5.5. Association Analysis of Genomic Regions Based on Permutation Tests

We used the R package regioneR [55] to answer two questions:

- Do the DMCs in one set overlap with those in another set more than expected by chance?
- Are the DMCs in one set significantly closer to those in the other set?

This package statistically evaluates association between two different region sets using a permutation-based approach. There are 3 main elements needed to perform the permutation test: two region sets, a randomization strategy and an evaluation function. In our case, the association was evaluated between two sets of DMCs. The built-in function resampleRegions was used as the randomization strategy to randomly select a subset from the “universe” to create the randomized DMC set. The sampling universe was set to all CpGs available to both Dataset 1 and Dataset 2, and the number of randomizations was set to 10,000. We can compare our evaluation from real data with those obtained randomly to determine whether it was plausible that our original evaluation was obtained by chance or not. Based on which question we were answering, the evaluation strategy was chosen from two built-in functions: numOverlaps and meanDistance.

### 5.6. Integration Studies with Data from GWAS Catalog

#### 5.6.1. Identifying SNPs in the GWAS Catalog Associated with RA

Genetic loci associated with RA were downloaded from GWAS Catalog web interface (https://www.ebi.ac.uk/gwas/efotraits/EFO_0000685 (accessed on 31 March 2021, accession number: EFO_0000685)). Non-SNP variants, variants not on autosomes and variants associated with child traits of RA (ACPA-negative RA, ankylosing spondylitis, psoriatic arthritis) were excluded from analysis. Duplicates and variants with reported *p*-values $>5\times 10^{-8}$ were also removed.

#### 5.6.2. Linking DMRs and SNPs to Annotated Protein-Coding Genes

The gene annotations for DMRs based on genomic features were obtained by using R package annotatr [56]. The genomic features of a DMR were extracted from org.Hs.eg.db [57]. Features obtained included whether a DMR was located in the 1–5 kbp upstream of TSS, the promoter or the gene body (with at least one base pair overlap) of a protein-coding gene. Protein-coding gene IDs and gene symbols were provided by TxDb.Hsapiens.UCSC.hg19.knownGene [58].

The mapped gene information for SNPs was obtained from the GWAS Catalog directly.

#### 5.6.3. Gene Ontology Analysis and Canonical Pathway Information

Gene ontology analysis detects significant over-representation of GO terms in one set with respect to a chosen background set. The analyses for each gene set were determined by the function gost from R package gprofiler2 [17] [database version: Ensembl 103] with default parameters, and the gene set from the immune panel was used as the background set. GO term were extracted from BioMart release 1 February 2021, the GO terms with g:SCS multiple testing corrected *p*-value <0.05 are considered as significant.

Canonical pathway information was extracted from the database of QIAGEN IPA software [Version 0-19-02].

## Figures and Tables

**Table 1:** Demographics of participants in initial study (Dataset 1) and current replication study (Dataset 2).

	Dataset 1				Dataset 2			
	**All Subjects** **(** n=121 **)**	**ACPA-Pos** **(** n=54 **)**	**ACPA-Neg** **(** n=61 **)**	**RA** **(** n=6 **)**	**All Subjects** **(** n=112 **)**	**ACPA-Pos** **(** n=50 **)**	**ACPA-Neg** **(** n=54 **)**	**RA** **(** n=8 **)**
ACPA OD, mean (range)	39.0 (2.8–228.5)	65.0 (40.1–210.4)	7.0 (2.8–19.0)	133.5 (4.4–228.5)	55.8 (3.5–191.6)	98.3 (60.2–178.9)	7.3 (3.5–18.8)	117.0 (3.5–191.6)
Age, mean (sd)	54.8 (7.7)	54.8 (8.0)	54.7 (7.5)	55.3 (9.5)	54.2 (7.9)	54.4 (7.7)	54.2 (8.1)	53.0 (8.1)
Female, n (%)	78 (64.5)	33 (61.1)	39 (63.9)	6 (100)	62 (55.4)	29 (58)	30 (55.6)	3 (37.5)
Smoker, n (%)								
Current	26 (21.5)	12 (22.2)	13 (21.3)	1 (16.7)	18 (16.1)	6 (12)	10 (18.5)	2 (25)
Past	47 (38.8)	23 (42.6)	22 (36.1)	2 (33.3)	48 (42.9)	20 (40)	24 (44.4)	4 (50)
Never,	4 (3.3)	0 (0)	4 (6.6)	0 (0)	4 (3.6)	3 (6)	1 (1.9)	0 (0)
Missing	44 (36.4)	19 (35.2)	22 (36.1)	3 (50)	42 (37.5)	21 (42)	19 (35.2)	2 (25)
Blood cell proportions,	mean (range)							
monocyte	0.077 (0.022)	0.077 (0.020)	0.077 (0.023)	0.079 (0.040)	0.079 (0.019)	0.079 (0.019)	0.081 (0.019)	0.075 (0.019)
lymphocyte	0.280 (0.068)	0.281 (0.072)	0.280 (0.063)	0.280 (0.096)	0.286 (0.072)	0.283 (0.069)	0.299 (0.067)	0.219 (0.093)
neutrophil	0.613 (0.078)	0.614 (0.079)	0.612 (0.071)	0.615 (0.140)	0.604 (0.082)	0.607 (0.082)	0.590 (0.076)	0.680 (0.093)
eosinophil	0.023 (0.015)	0.023 (0.017)	0.024 (0.014)	0.020 (0.008)	0.025 (0.017)	0.026 (0.016)	0.025 (0.019)	0.021 (0.012)
basophil	0.007 (0.004)	0.007 (0.005)	0.006 (0.004)	0.008 (0.004)	0.006 (0.004)	0.006 (0.004)	0.005 (0.004)	0.004 (0.003)

Note: subjects without cell-type composition information were removed for both datasets. One subject in Dataset 1 with low-positive ACPA level (OD = 29.67) was also removed, leaving only medium-positive (40 < OD ≤ 60) and high-positive (OD > 60) ones for the ACPA-positive group in Dataset 1. All subjects in the positive group of Dataset 2 were highly positive (OD > 60). Abbreviations: ACPA: anti-citrullated protein antibodies; RA: rheumatoid arthritis; OD: optical density.

**Table 2:** Number of CpGs covered in the two datasets, and overlaps in captured sites.

	Dataset 1	Dataset 2	Overlaps
# of CpGs covered in at least two samples with at least one read	5,041,032	5,307,142	3,948,157
# of CpGs covered after quality control	1,305,080	4,259,820	1,095,002

**Table 3:** Summary of different models fitted and corresponding identified DMCs/DMRs.

Models	#CpGs Tested	#DMCs (#DMRs)	#HyperDMCs (#HyperDMRs)	#HypoDMCs (#HypoDMRs)
I. ACPA-positive vs. ACPA-negative (Dataset 2)	4,259,820	19,472 (814)	8581 (334)	10,891 (480)
II. ACPA-positive vs. ACPA-negative (Dataset 1)	1,305,080	853 (44)	569 (31)	284 (13)
Overlaps by position	1,095,002	157 (10)	43 (3)	16(1)
III. ACPA-positive vs. ACPA-negative with genetic effect adjustment (Dataset 2)	19,472 *	6314 (302)	2415 (115)	3899 (187)
IV. ACPA-positive vs. ACPA-negative with genetic effect adjustment (Dataset 1)	853 ^†^	515 (28)	371 (22)	144 (6)
Overlaps by position	157	31 (3)	14 (1)	1 (0)
V. Self-reported RA vs. Asymptomatic (Dataset 2)	4,282,792	18,874 (843)	10,909 (578)	7965 (265)
VI. Self-reported RA vs. Asymptomatic (Dataset 1)	1,295,623	258 (15)	99 (5)	159 (10)
Overlaps by position	1,099,279	55 (4)	15 (1)	11 (1)

* In which 6026 DMCs found not to be affected by cis-SNPs were refitted without genetic adjustment. † In which 503 DMCs found not to be affected by cis-SNPs were refitted without genetic adjustment.

**Table 4:** The number of DMCs (DMRs) identified in the initial and this replication study, and overlap in captured sites.

	Initial Study (Dataset 1)	Replication Study (Dataset 2)	Overlaps	Overlaps (Consistent)
ACPA-positive vs. ACPA-negative				
# of CpGs tested	4,635,909	4,259,820		
# of DMCs(DMRs) identified	1909 (509)	19,472 (814)	410 (23)	230 (11)
Self-reported RA vs. ACPA asymptomatic				
# of CpGs tested	4,109,916	4,282,792		
# of DMCs(DMRs) identified	955 (249)	18,874 (843)	156 (9)	110 (6)

**Table 5:** Summary of protein-coding genes associated with identified ACPA and RA DMRs, and those associated with RA causal SNPs from GWAS Catalog.

Source	# of Mapped Genes	Overlap with GWAS Genes
585 SNPs from GWAS Catalog	295	
814 ACPA-associated DMRs	403	*HLA-DRB1*, *HLA-DRB5* *ERICH1*, *KIF26B*, *SPAG1* *DUSP22*, *DOCK1*, *NTM* *DGKQ*, *PRDM16*, *RAD51B* *TP73*, *SLC9A9*, *ZNF595*
843 RA-associated DMRs	376	*ERICH1*, *ZNF595*, *SPAG1*, *TP73* *PADI4*, *CARD9*, *CTIF*

**Table 6:** Gene Ontology terms that collectively show over-representation in all gene sets, at corrected *p*-value <0.05.

Source	Term ID	Term Name	*p*-Value (GWAS)	*p*-Value (ACPA-DMR)	*p*-Value (RA-DMR)
GO:BP	GO:0098609	cell–cell adhesion	$3.7\times 10^{-8}$	$4.2\times 10^{-17}$	$1.0\times 10^{-12}$
GO:BP	GO:0007155	cell adhesion	$3.5\times 10^{-6}$	$8.4\times 10^{-14}$	$2.9\times 10^{-9}$
GO:BP	GO:0022610	biological adhesion	$4.1\times 10^{-6}$	$1.1\times 10^{-13}$	$3.6\times 10^{-9}$
GO:CC	GO:0005886	plasma membrane	$4.1\times 10^{-2}$	$8.7\times 10^{-9}$	$8.8\times 10^{-3}$

**Table 7:** Gene symbols involved in cell–cell adhesion (GO:0098609) for each of the profiled gene sets, with the number of associated SNPs/DMRs.

Gene List	Genes Involved	#SNPs/DMRs Associated
GWAS Catalog	*PTPN22, CYRIB, SRC, HLA-DQB1, ETS1, ZFP36L1, PTPN2* *RUNX1, TNFSF4, IL2RA, PRKCQ, GATA3, SWAP70, BAD* *CD83, BLK, CTLA4, ICOS, CXCL13, SH2B3, CCR2, CD28* *TNIP1, HLA-DRB1, CDH18, NOTCH4, PCDH15, PTPRM* *SFTPD, FCGR2B, IL2, IL21, TRAF6, DLG2, RASGRP1* *HLA-DRA, PTPRC, CD200R1, KIF26B, DPP4, ICAM3* *PTPN11, EMCN*	86
ACPA-associated DMRs	*HLA-DRB1, MDGA1, C1QTNF1, AP3D1, CLSTN1, CNTN4* *CELSR3, ALOX12, SCRIB, CRB2, PKP3, MAG, PLPP3, KIF26B* *PDGFRA, MAD1L1, SDK1, CDHR3, DSCAML1, UBASH3B* *JAM3, SDK2, MBP, PCDHA8, PCDHB8, PCDHB16, PCDHB10* *PCDHB13, PCDHB15, PCDHGB3, PCDHGA12, PCDHA13* *PCDHA2, PCDHA7, PCDHB3, PCDHB4, PCDHB5, PCDHB6* *PCDHB7, PCDHB11, PCDHB12, PCDHGA1, PCDHGA5* *PCDHA1, PCDHA3, PCDHA4, PCDHA5, PCDHA6, PCDHA9* *PCDHA10, PCDHA11, PCDHA12, PCDHGA2, PCDHGA3* *PCDHGB1, PCDHGA4, PCDHGA7, PCDHGB4, PCDHGA8* *PCDHGB5, PCDHGA6, PCDHGA9, PCDHGB6, PCDHGA10* *PCDHGB2, PCDHGB7, PCDHGA11*	55
RA-associated DMRs	*CD160, FGL1, RDX, PIEZO1, AP3D1, MEGF10, SCRIB, ITGA8* *PKP3, LRRC32, MAG, CELSR1, PRKCZ, GLI2, NCK1, PDGFRA* *MAD1L1, SDK1, CDHR3, UBASH3B, GPC6, NTN1, MBP, PAK4* *CDH4, RIPOR2, PCDHA3, PCDHB3, PCDHB8, PCDHB16* *PCDHGA3, PCDHGB1, PCDHGB2, PCDHGB3, PCDHGA8* *PCDHGB4, PCDHA2, PCDHA12, PCDHB7, PCDHB10, PCDHB11* *PCDHB13, PCDHB14, PCDHGA1, PCDHGA2, PCDHGA4* *PCDHGA6, PCDHGA7, PCDHA1, PCDHA4, PCDHA5, PCDHA6* *PCDHA7, PCDHA8, PCDHA9, PCDHA10, PCDHA11, PCDHGA5*	49

## Data Availability

Results from this study will be returned to CARTaGENE, all relevant results and data are available on request from this resource (https://www.cartagene.qc.ca/en/researchers (accessed on 24 July 2021)).

## Supplementary Materials

The following are available at https://www.mdpi.com/article/10.3390/genes12091349/s1. Supplementary File 1: Table S1: results of DMCs from ACPA positive vs. negative comparison in the replicated dataset (Dataset 2), Table S2: results of DMCs from ACPA positive vs. negative comparison in the initial dataset (Dataset 1), Table S3: results of gDMCs from ACPA positive vs. negative comparison after genetic adjustment (Dataset 2), Table S4: results of gDMCs from ACPA positive vs. negative comparison after genetic adjustment (Dataset 1), Table S5: results of DMCs from comparison of RA patients vs. healthy subjects in the replicated dataset (Dataset 2), Table S6: results of DMCs from comparison of RA patients vs. healthy subjects in the initial dataset (Dataset 1), Table S7: results of overlapped ACPA-associated DMCs by comparison with the initial study (Model I vs. published), Table S8: results of overlapped RA-associated DMCs by comparison with the initial study (Model V vs. published), Table S9: Gene Ontology terms showing over-representation with corrected *p*-values < 0.05, for the list of genes derived from the ACPA-associated DMRs, Table S10: Gene Ontology terms showing over-representation with corrected *p*-values < 0.05, for the list of genes derived from the RA-associated DMRs, Table S11: Gene Ontology terms showing over-representation with corrected *p*-values < 0.05, for the list of genes derived from the RA GWAS loci. Supplementary File 2: RA-associated GWAS loci downloaded from GWAS Catalog on 31 March 2021.
